# THE INTEGRATED MEMORY CARE CLINIC AS A HEALTHCARE NETWORK

**DOI:** 10.1093/geroni/igz038.2067

**Published:** 2019-11-08

**Authors:** Mariya A Kovaleva, Melinda Higgins, Bonnie Mowinski Jennings, Mi-Kyung Song, Carolyn K Clevenger, Patricia C Griffiths, Kenneth W Hepburn

**Affiliations:** 1 Vanderbilt University School of Nursing, Nashville, Tennessee, United States; 2 Nell Hodgson Woodruff School of Nursing Emory University, Atlanta, Georgia, United States; 3 Atlanta VA Medical Center, Center for Visual and Neurocognitive Rehabilitation, Decatur, Georgia, United States

## Abstract

The Integrated Memory Care Clinic (IMCC) at Emory Healthcare is a patient-centered medical home led by advanced practice registered nurses (APRNs) who provide dementia and primary care. This presentation describes the experiences of persons living with dementia and their caregivers during their first year at the IMCC, through the lens of the IMCC as a healthcare network. Forty-two caregivers were evaluated in three survey-based assessments over nine months. Twelve caregivers completed qualitative interviews about their experience at the IMCC. Severity of depression and delusions and total symptom severity improved significantly for persons living with dementia. Caregivers described their sense of belonging to the IMCC healthcare team and valued direct telephone access to APRNs. By enhancing care access and engaging clients in their care, the IMCC serves as a reliable and professional healthcare network for patient-caregiver dyads who often receive suboptimal dementia care in mainstream healthcare.

